# *Clostridium acetobutylicum* grows vegetatively in a biofilm rich in heteropolysaccharides and cytoplasmic proteins

**DOI:** 10.1186/s13068-018-1316-4

**Published:** 2018-11-20

**Authors:** Dong Liu, Zhengjiao Yang, Yong Chen, Wei Zhuang, Huanqing Niu, Jinglan Wu, Hanjie Ying

**Affiliations:** 1 State Key Laboratory of Materials-Oriented Chemical Engineering, College of Biotechnology and Pharmaceutical Engineering, Nanjing Tech University, No. 30, Puzhu South Road, Nanjing, 211800 China; 2 Jiangsu National Synergetic Innovation Center for Advance Material (SICAM), No. 30, Puzhu South Road, Nanjing, 211800 China

**Keywords:** *Clostridium acetobutylicum*, Biofilm, Polysaccharide, Moonlighting protein, Sporulation

## Abstract

**Background:**

Biofilms are cell communities wherein cells are embedded in a self-produced extracellular polymeric substances (EPS). The biofilm of *Clostridium acetobutylicum* confers the cells superior phenotypes and has been extensively exploited to produce a variety of liquid biofuels and bulk chemicals. However, little has been known about the physiology of *C. acetobutylicum* in biofilm as well as the composition and biosynthesis of the EPS. Thus, this study is focused on revealing the cell physiology and EPS composition of *C. acetobutylicum* biofilm.

**Results:**

Here, we revealed a novel lifestyle of *C. acetobutylicum* in biofilm: elimination of sporulation and vegetative growth. Extracellular polymeric substances and wire-like structures were also observed in the biofilm. Furthermore, for the first time, the biofilm polysaccharides and proteins were isolated and characterized. The biofilm contained three heteropolysaccharides. The major fraction consisted of predominantly glucose, mannose and aminoglucose. Also, a great variety of proteins including many non-classically secreted proteins moonlighting as adhesins were found considerably present in the biofilm, with GroEL, a S-layer protein and rubrerythrin being the most abundant ones.

**Conclusions:**

This study evidenced that vegetative *C. acetobutylicum* cells rather than commonly assumed spore-forming cells were essentially the solvent-forming cells. The abundant non-classically secreted moonlighting proteins might be important for the biofilm formation. This study provides the first physiological and molecular insights into *C. acetobutylicum* biofilm which should be valuable for understanding and development of the biofilm-based processes.

**Electronic supplementary material:**

The online version of this article (10.1186/s13068-018-1316-4) contains supplementary material, which is available to authorized users.

## Background

Microbial cells could synthesize extracellular polymeric substances (EPS) to build biofilm communities with enhanced survival and metabolic capacities [1]. In natural settings, biofilms are commonly formed by cells attached to surfaces or interfaces, like the surfaces of water pipes, stones in a river, and indwelling devices in hospital patients. In laboratory, biofilms are usually attached to the inside wall of incubators, grown on agar plates or static liquid surfaces [2], and their formation could often be facilitated by solid carriers submerged in culture media, such as cotton fibre, plastic, stainless steel, glass or clay brick [3, 4]. Some nutritional factors were shown to be important for biofilm formation by some species. For some prokaryotes like *Staphylococcus aureus*, *Pseudomonas aeruginosa* and *P. fluorescens,* iron limitation repressed biofilm formation while high iron rescued biofilm formation [5, 6]. In *Escherichia coli*, *Salmonella* sp. and anaerobic sludge communities, low-nutrient media (e.g., glucose-minimum medium, minimum-salts medium, media with relatively less peptone or a high C/N ratio) appeared to favor EPS production and biofilm formation [7–9]. As a multicellular form of microbial life, biofilms could exhibit emergent properties that are quite distinct from those of free-living cells and have attracted increasing attention in biotechnological processes as well as in medical processes [1]. More and more biofilms are engineered as cell factories for biomanufacturing [10]. One canonical example is the biofilm of solventogenic *Clostridium acetobutylicum* which is an important industrial platform capable of producing a range of biofuels and bulk chemicals [11]. It was shown that butanol tolerance of *C. acetobutylicum* cells in biofilm was three orders of magnitude higher than that of planktonic cells under certain conditions [12]. Operated in a continuous mode, *Clostridium* biofilms increased the productivity by almost 50-fold [4, 13]. Enhanced metabolism of pentose as well as hexose and enhanced solvent biosynthesis were also extensively demonstrated for *C. acetobutylicum* biofilm [14–16].

In general, superior phenotypes (such as the improved tolerance and metabolic activities) of EPS-encased biofilm cells could be attributed to two aspects: genetic regulation and EPS architecture. Living together in biofilms, cells tend to exhibit a different pattern of gene expression. Some genes are repressed or activated, thus cellular structure and functions are modulated [17, 18]. On the other hand, highly hydrated EPS matrix can be a protective barrier and provides cells with a favourable microenvironment. EPS plays an important role in exclusion of toxic substances [17, 19]. It keeps cells in close proximity and enables the development of high-density cell communities with intense cell–cell communication and cooperation [19, 20]. EPS matrix also provides excellent conditions for retention of extracellular proteins, functioning as an enzyme reservoir for external processes [1].

However, so far little has been known about *C. acetobutylicum* biofilm. Despite the observation of *C. acetobutylicum* biofilm under various conditions, the underlying molecular basis and regulatory processes remain to be explored [21]. Deciphering the EPS matrix and cell physiology in the biofilm will be important for optimization and control of biofilm-based processes. Recently we reported the first transcriptomic study of *C. acetobutylicum* biofilm [22] and revealed that heterogeneity of *C. acetobutylicum* EPS conferred improved resistance to harsh environments [23]. In this study, we will further shed light on *C. acetobutylicum* biofilm by investigating the cell physiology and EPS composition in the biofilm.

## Methods

### Culture and medium

Cultures of *C. acetobutylicum* B3 (CGMCC 5234) were grown in modified P2 medium containing 10 g/L glucose as the sole carbohydrate source for seed culture. Fermentation experiments were performed anaerobically in 2-L stainless steel columns containing 1.5 L of P2 medium (glucose 60 g/L; K_2_HPO_4_ 0.5 g/L; KH_2_PO_4_ 0.5 g/L; CH_3_COONH_4_ 2.2 g/L; MgSO_4_·7H_2_O 0.2 g/L; MnSO_4_·H_2_O 0.01 g/L; NaCl 0.01 g/L; FeSO_4_·7H_2_O 0.01 g/L; *p*-aminobenzoic acid 1 mg/L; thiamine 1 mg/L; biotin 0.01 mg/L) at 37 °C with initial inoculum 10%(v/v). Cotton towel was used to facilitate the formation of biofilm and continuous culture was performed with broth replacement every 12 h, see our previous work for details [12, 22].

### Quantification of biofilm formation

Each piece of cotton towel (2 cm × 3 cm) with attached biofilm was taken from fermenters at predetermined time, immersed in 20 mL of 0.1 M NaOH (the mass of NaOH solute was calculated as* W*1) and vortexed to completely dissolve the biofilm. Then, the piece was removed and rinsed twice with a total of 40 mL of pure water. All the volumes were mixed together and the total volume was determined. Then, 3 mL of the mixture was dried and weighed to deduce the total dry weight (*W*2) of the mixture. Biofilm formation was quantified as (*W*2 − *W*1)/(2 cm × 3 cm).

### Transcriptomic analysis

To collect biofilm cells for transcriptomic analysis, pieces of cotton towel were harvested from the fermenter typically at 6 h after each broth replacement and rinsed twice with PBS buffer (137 mM NaCl, 2.7 mM KCl, 8 mM Na_2_HPO_4_ and 2 mM KH_2_PO_4_, pH 7.4, 4 °C) to remove contaminating planktonic cells. Then, the cotton towel was submerged in 15-mL PBS buffer and the biofilm was scraped off the cotton towel. The resulting suspension was centrifuged at 8000×*g* for 6 min at 4 °C to pellet the biofilm cells. All the cells were frozen immediately using liquid nitrogen and then stored at − 80 °C prior to RNA extraction. RNA extraction and transcription analysis were performed as previously described [22]. Resulting microarray data were uploaded to the Gene Expression Omnibus (GEO) database under Accession Number GSE72765. Hierarchical clustering was performed using R-software and clusters were visualized with Tree-view [22].

### Microscopy

Light microscopy was used for morphological observation. Each piece of cotton towel (approximately 2 cm × 3 cm) with attached biofilm was taken from fermenters at predetermined time points, immersed in 0.1 M NaOH at 4 °C for 10 min and vortexed to completely disperse cells within the biofilm. Then, 50 ul of suspension was transferred to a microscope slide and air-dried. Safranin O was used as a fluorescence dye that can be excited with green light [24]. It is well known for nuclear staining and was also reported to stain mucin, cartilage, starch and plant tissues [25]. In this study, it also differentially stained cells and endospores under light microscopy. Cells were stained with 0.5% safranin O for 30 s, washed gently with water and then air-dried for light microscopy. As the real production system with cotton towel as biofilm carrier was not suitable for detailed observation of the biofilm, fluorescence microscopy using microscope slides as biofilm carriers was used to observe the biofilm in detail. Microscope slides were immersed in 100-mL Duran bottles containing 50 mL of fermentation broth at the time of inoculation (10%, v/v). All the bottles were kept static in an anaerobic system (Whitley DG250 workstation, Don Whitley Scientific Limited, UK) at 37 °C. After predetermined time intervals, the slides were withdrawn from the bottles, rinsed twice with PBS buffer (pH 7.4), then air-dried in the anaerobic system. Samples were stained with 0.5% safranin O as described above before imaging in the green channel in a Leica DM2500 microscope.

### Extraction of polysaccharides and proteins from *C. acetobutylicum* biofilm

Various methods were tried to dissolve *C. acetobutylicum* biofilm, including EDTA treatment, hot water treatment, sonication and enzymatic digestion. Usually, these methods could physically or chemically dissolve biofilm matrixes and have been commonly investigated for EPS extraction [26]. However, none of these methods could effectively dissolve the *C. acetobutylicum* biofilm except using NaOH (Additional file 1: Figure S1). The supernatant proteins and polysaccharides extracted by NaOH were much more than those from other methods, while the DNA ratio which could be an indicator of cell lysis was adequately low (Additional file 1: Table S1). Biofilm attached on cotton towel was immersed in 0.1 M NaOH at 4 °C for 30 min. This would get the biofilm matrix dissolved completely and quickly. The suspension was then centrifuged (10,000*g*) at 4 °C for 10 min to pellet cells. To the supernatant containing soluble EPS, 1.5 volume of ethanol was added to precipitate polysaccharides. Precipitated polysaccharides were collected through centrifugation and the supernatant was adjusted to pH 4.2 and kept overnight at 4 °C to precipitate proteins. Interference of possible cell lysis on the process was excluded because cells were all apparently intact after the short treatment with NaOH as was confirmed by microscopy. Also, control experiments solely with an equal volume of *C. acetobutylicum* cells going through the same procedures did not yield apparent sediments.

### Isolation of polysaccharides

An aliquot of 0.5 g wet polysaccharide extract was dissolved in 35 mL of 0.1 M NaOH. The polysaccharide solution was filtered through a 0.45-µm membrane filter (Fisher Scientific). Polysaccharides were isolated with the Q-Sepharose fast flow (QFF) chromatography column (AKTA, GE Healthcare, USA), eluted with a step gradient of NaCl (dissolved in 0.1 M NaOH) in 0, 0.3, 0.4, 0.6, and 0.8 M steps, at a flow rate of 1.5 mL/min. Eluent was monitored at 280 nm and carbohydrate content of each fraction (200 µL) was determined according to the phenol–sulphuric acid method [27].

### Monosaccharide composition analysis

The 1-phenyl-3-methyl-5-pyrazolone (PMP) derivatization method [28] was used to analyze monosaccharide composition. Each polysaccharide (2 mg) was hydrolyzed with 2 M trifluoroacetic acid (TFA) at 105 °C for 3 h. TFA was evaporated by adding methanol under reduced pressure. The hydrolysis product was derivatized with PMP in 0.3 M NaOH for 1 h at 70 °C, and then neutralized with HCl. The derivatives were analyzed using a Thermo C18 column (250 mm × 4.6 mm) coupled to an Agilent HPLC–DAD at 245 nm, at a flow rate of 0.8 mL/min of mobile phase: phosphate buffer (0.1 mol/L, pH 7.0)/CH_3_CN = 83/17 (v/v). Monosaccharide composition and the molar ratio analysis were carried out by comparing the retention times and peak areas with those of monosaccharide standards.

### Mass spectrometric analysis of extracellular proteins

Proteins precipitated after the pH adjustment were sent to Shanghai Boyuan Biological Technology CO., LTD (Shanghai, China) for LC–MS/MS analysis. Proteins isolated by two-dimensional (2D) SDS-PAGE were analyzed using an ABI 4800 Plus MALDI TOF/TOF system (Life Technologies). Protein identification was performed using MASCOT 2.3 (http://www.matrixscience.com/, Matrix Science, UK) against the NCBI-*Clostridium acetobutylicum* database using a significance threshold of* p *< 0.05.

## Results

### Sporulation and morphological changes of biofilm cells

**Fig. 1:**
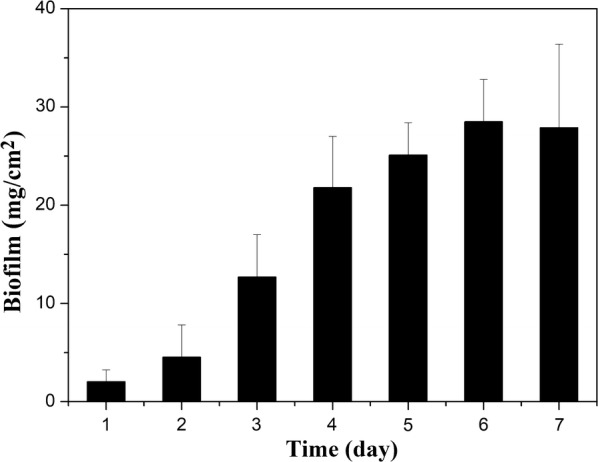
Amounts of biofilm accumulated on cotton towel over time

**Fig. 2:**
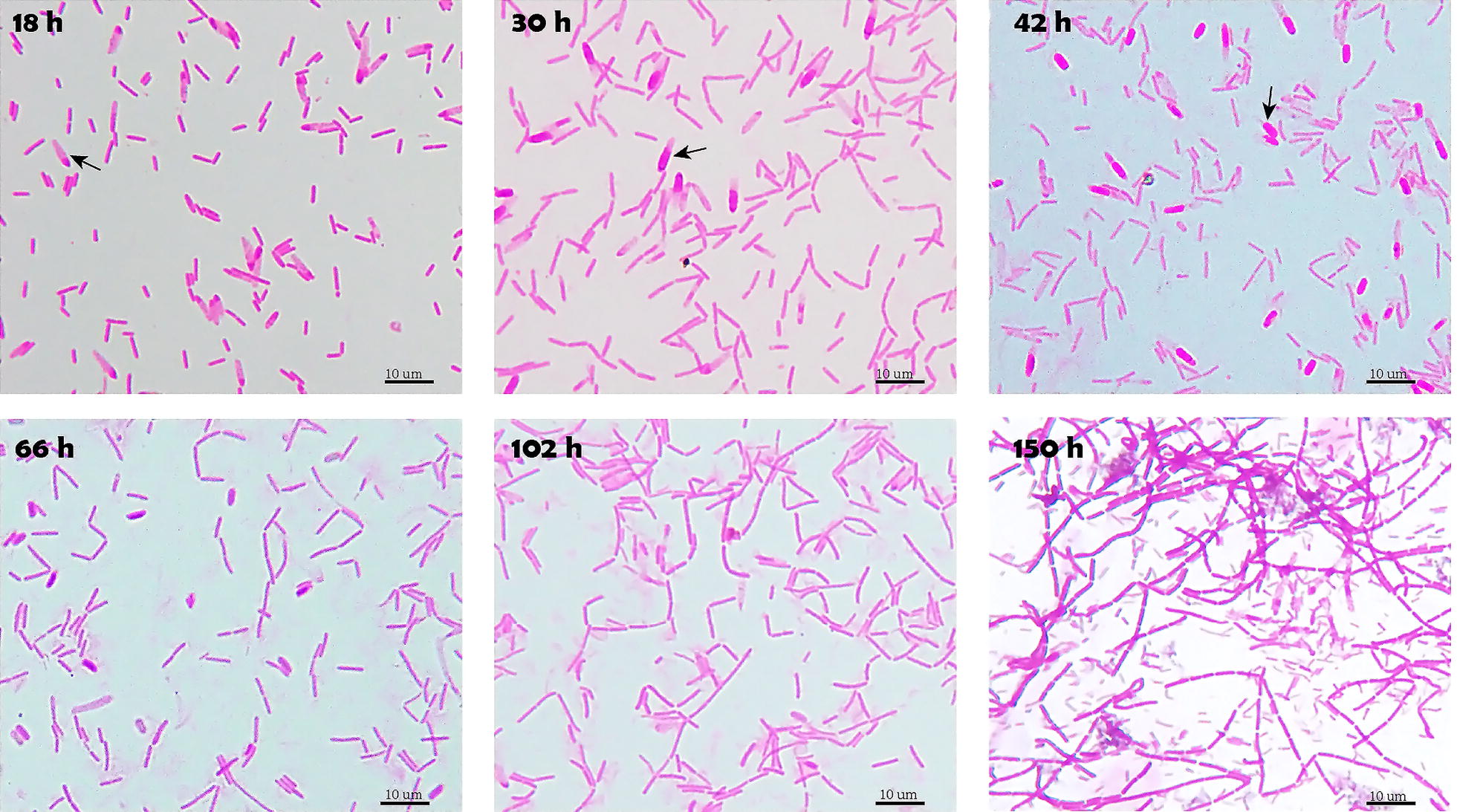
Elimination of sporulation and vegetative growth of *C. acetobutylicum* in the biofilm over time. The arrow indicates the clostridial-form cell (18 h), forespore (30 h) or free spore (42 h)

Biofilm formation by *C. acetobutylicum* in fermenters during continuous cultivation was quantified. Accumulation of biofilm was most apparent during day 3 and day 4. The biofilm could be built up with a maximum dry weight of 28 mg/cm^2^ on the surface of cotton towel (Fig. 1). It was found that during continuous cultivation of *C. acetobutylicum* biofilm, the cells eventually eliminated sporulation and displayed a vegetative growth. As shown in Fig. 2, swollen clostridial-form cells first appeared at 18 h. These cells are to be the mother cells for spores. With the sporulation, fore spores were formed at 30 h. The final mature spores were released and peaked at 42 h, after which they disappeared rapidly. By 102 h, spores could hardly be observed, leaving almost exclusively vegetative cells. At the same time, the vegetative cells underwent significant morphological changes: from short single cells to long-chain cells. The long chains of cells were observed from 66 h, apparent at 102 h, and predominantly present in the biofilm after 150 h with a length around 100 µm.

### Decreased expression of sporulation genes in biofilm cells

**Fig. 3:**
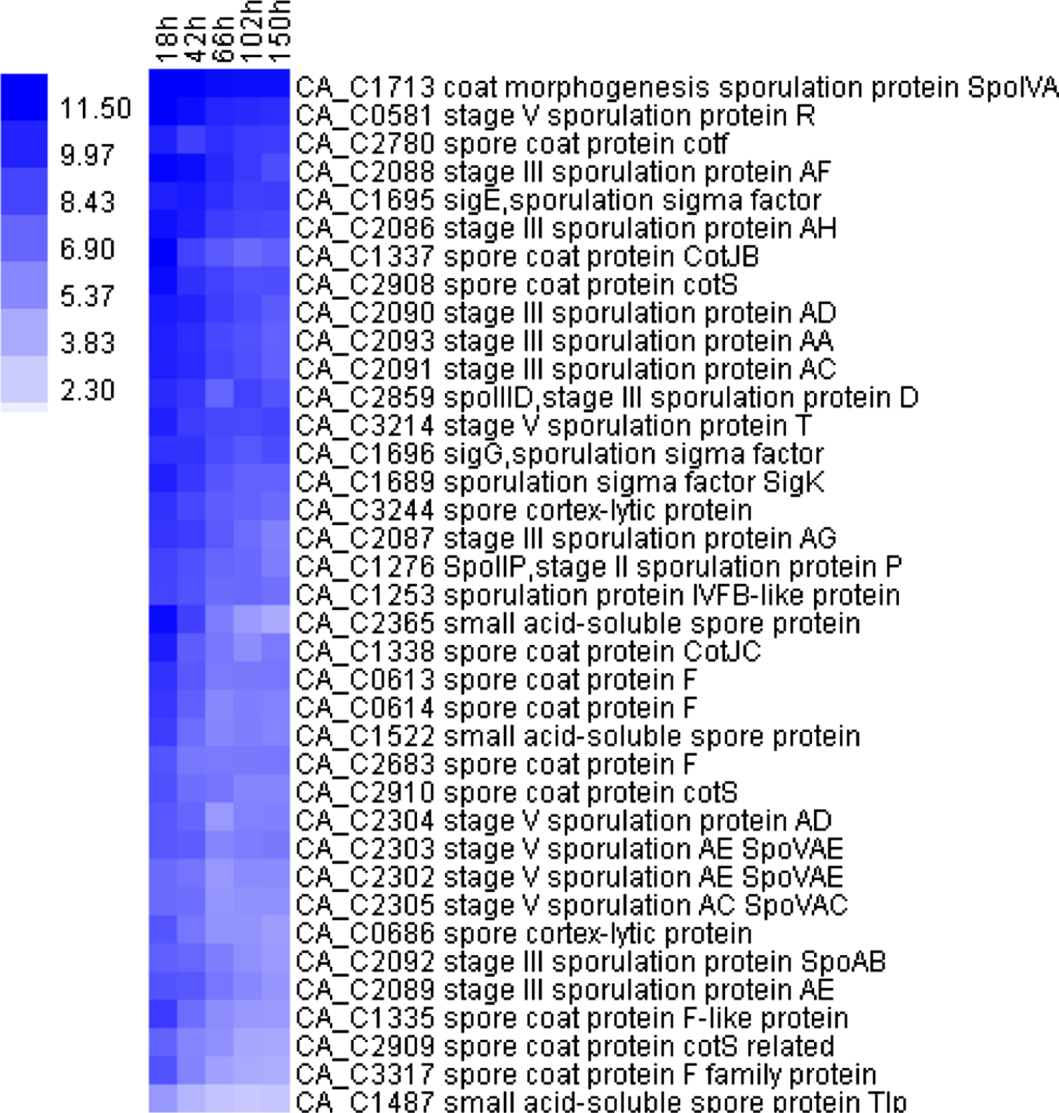
Temporal expression of sporulation genes in *C. acetobutylicum* biofilm cells. The values on the color bar represent log (base 2) transformed gene expression levels

Inspired by the elimination of sporulation in the biofilm cells during long-term cultivation, expression of sporulation-related genes was investigated. In general, expression of the genes responsible for spore formation was apparently down-regulated in the biofilm over time (Fig. 3). Of the sporulation regulators in *C. acetobutylicum*, the gene encoding σ^K^ (*sig*K, CA_C1689) was downregulated over time by eightfold. The most down-regulated genes were those involved in spore coat synthesis (CA_C0613-0614, CA_C1335, CA_C1337-1338, CA_C2808-2910, CA_C3317), which decreased over time by 6- to 24-fold. An operon CA_C2086-2093 related to stage III sporulation was down-regulated 6- to 12-fold. The small, acid-soluble proteins (SASP) that are used to coat DNA in spores (encoded by CA_C1487 and CA_C1522) were also significantly down-regulated by 48-fold (*p *< 0.01; Student t test), and the CA_C2365 was down-regulated by 200-fold. Overall, the decreased expression of sporulation-related genes over time was consistent with the elimination of sporulation in the biofilm.

### EPS and wire-like structures in *C. acetobutylicum* biofilm

**Fig. 4:**
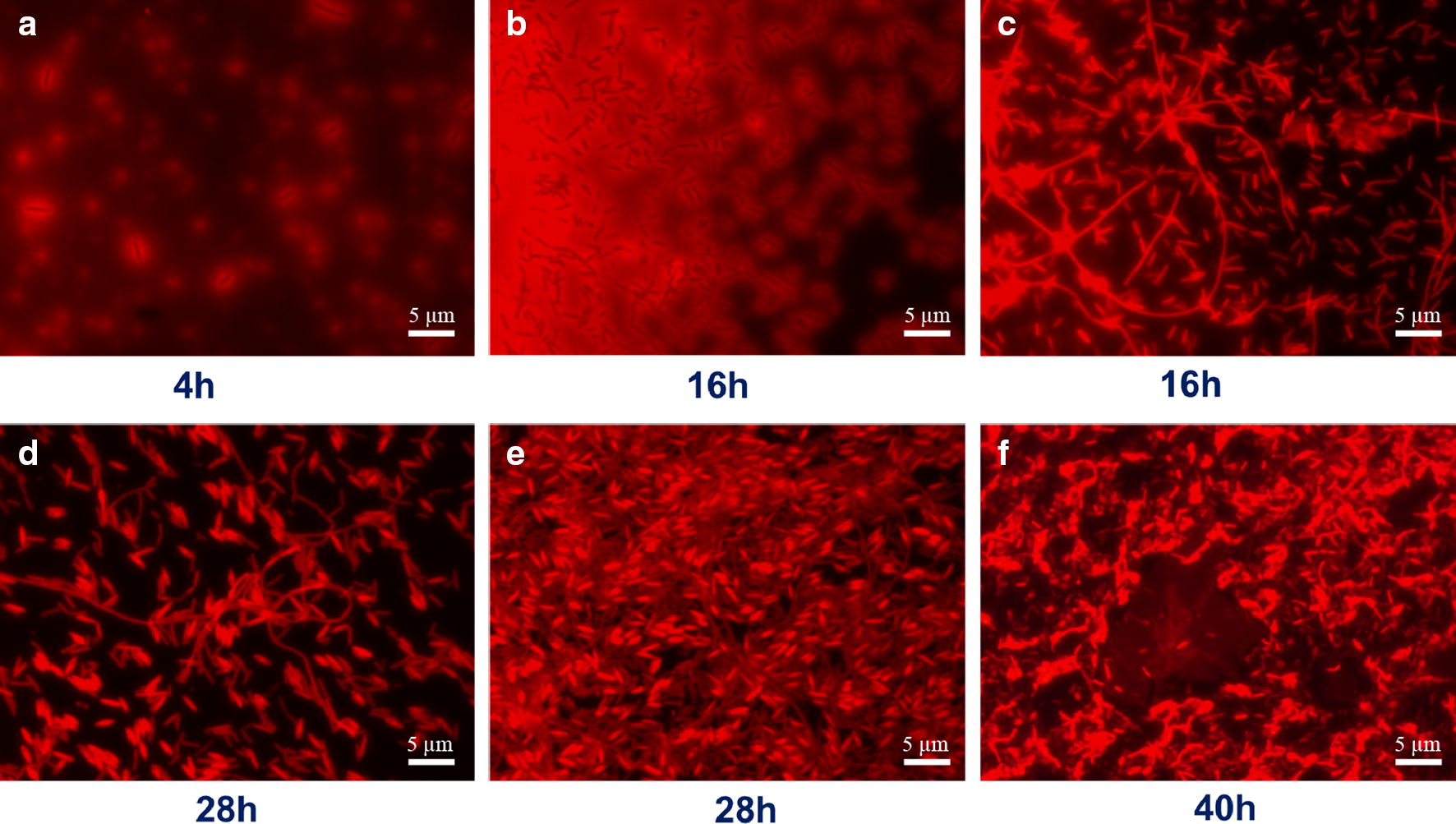
Extracellular polymeric substances and wire-like structures in *C. acetobutylicum* biofilm. Samples were stained with safranin O and imaged in a fluorescence microscope. **a** the early stage of biofilm development; **b** high-density cell colonies; **c** wire-like structures; **d** cellular morphology at the mid stage of biofilm development; **e** wires imbedded in cells aggregates; **f** EPS pellicles observed in the biofilm

EPS and wire-like structures of *C. acetobutylicum* biofilm were observed. As shown in Fig. 4, at the early stage of biofilm development (4 h), cells were all buried in a gel-like extracellular matrix (as indicated by the blurred area in the image) which was typically excreted by the cells to help adhere onto surfaces. Then it developed into a three-dimensional, high-density cell colony (16 h). At the edges of the colonies, wire-like structures could be clearly observed. The wires were surprisingly long (could be more than 50 µm) and could be cross-connected. With the development, the wires were eventually imbedded in cells aggregates (28 h). At the late stage (40 h), EPS pellicles were also shown. In recent years, a kind of “nanowire” structure has been reported for some bacterial biofilms and was supposed to function in extracellular electron transport [29]. Whether the wire structures observed here are similar to the nanowires and what their roles are in *C. acetobutylicum* biofilm remain to be investigated.

### Characterization of *C. acetobutylicum* biofilm polysaccharides

**Fig. 5:**
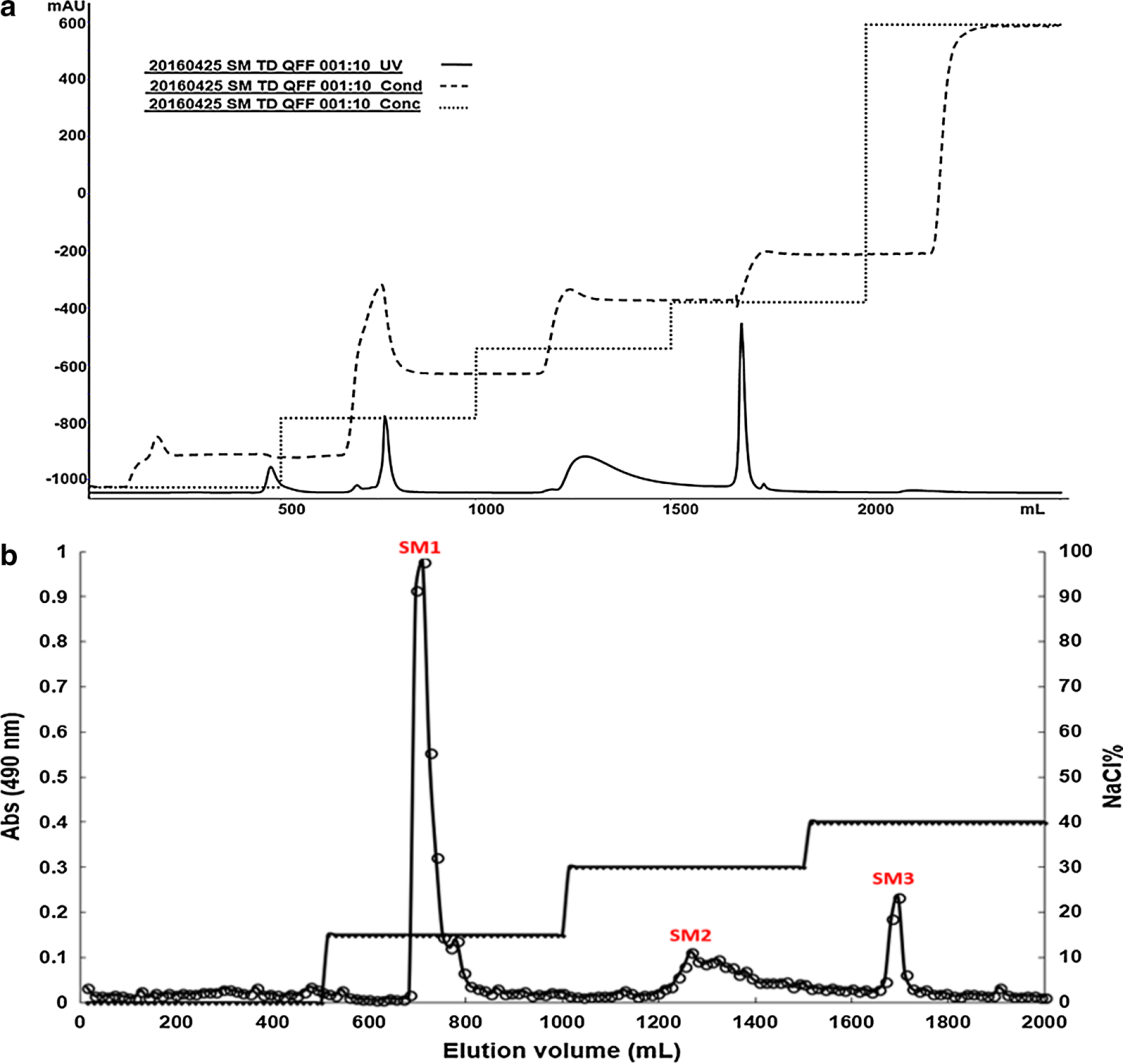
Isolation of extracellular polysaccharides produced by *C. acetobutylicum* biofilm on the QFF anion exchange column. **a** Elution profile monitored at 280 nm; **b** the profile of polysaccharides monitored at 492 nm by the phenol–sulfuric acid method

After polysaccharides and proteins in *C. acetobutylicum* biofilm were extracted, the polysaccharides were further isolated by anion exchange chromatography (Fig. 5). Three polysaccharides were obtained which were designated SM1, SM2, and SM3 according to their elution order. A peak at 280 nm occurred concurrently with each of the polysaccharide peaks, indicating possible presence of polysaccharide-associated proteins. The SM1 comprised the largest fraction (53%, w/w) of the polysaccharides, followed by SM2 (26%, w/w) and SM3 (21%, w/w). SM1 was also the most purified polysaccharide as indicated by a much smaller peak at 280 nm (Fig. 5).

Analysis of monosaccharide composition showed that all the three polysaccharides were heteropolysaccharides with glucose as the primary component (Table 1). SM2 and SM3 were very similar in both of monosaccharide type and molar ratio, consisting of glucose (47–53%, molar ratio, the same hereinafter), mannose (13–15%), rhamnose (10–16%), galactose (9–10%), aminoglucose (7–9%), and a little ribose (4–5%). Compared to SM2 and SM3, the SM1 polysaccharide consisted of more glucose (58%), mannose (21%), and aminoglucose (13%), but much less rhamnose (0.8%), galactose (0.8%) and ribose (0.4%). SM1 also consisted of unique galacturonic acid (5.5%). The presence of uronic acid might explain why the *C. acetobutylicum* biofilm matrix was alkali soluble.

**Table 1:** The molar ratio of each monosaccharide in *C. acetobutylicum* biofilm polysaccharides

	Glc	Man	GlcN	GalA	Rha	Gal	Rib
SM1	100	36	23	9.4	1.5	1.4	0.7
SM2	100	25	17	–	18	20	9.0
SM3	100	32	16	–	33	20	9.6

SM1, SM2 and SM3 are three isolated polysaccharides

*Glc* glucose, *Man* mannose, *GlcN* aminoglucose, *GalA* galacturonic acid, *Rha* rhamnose, *Gal* galactose; *Rib* ribose

### Identification of *C. acetobutylicum* biofilm proteins

**Fig. 6:**
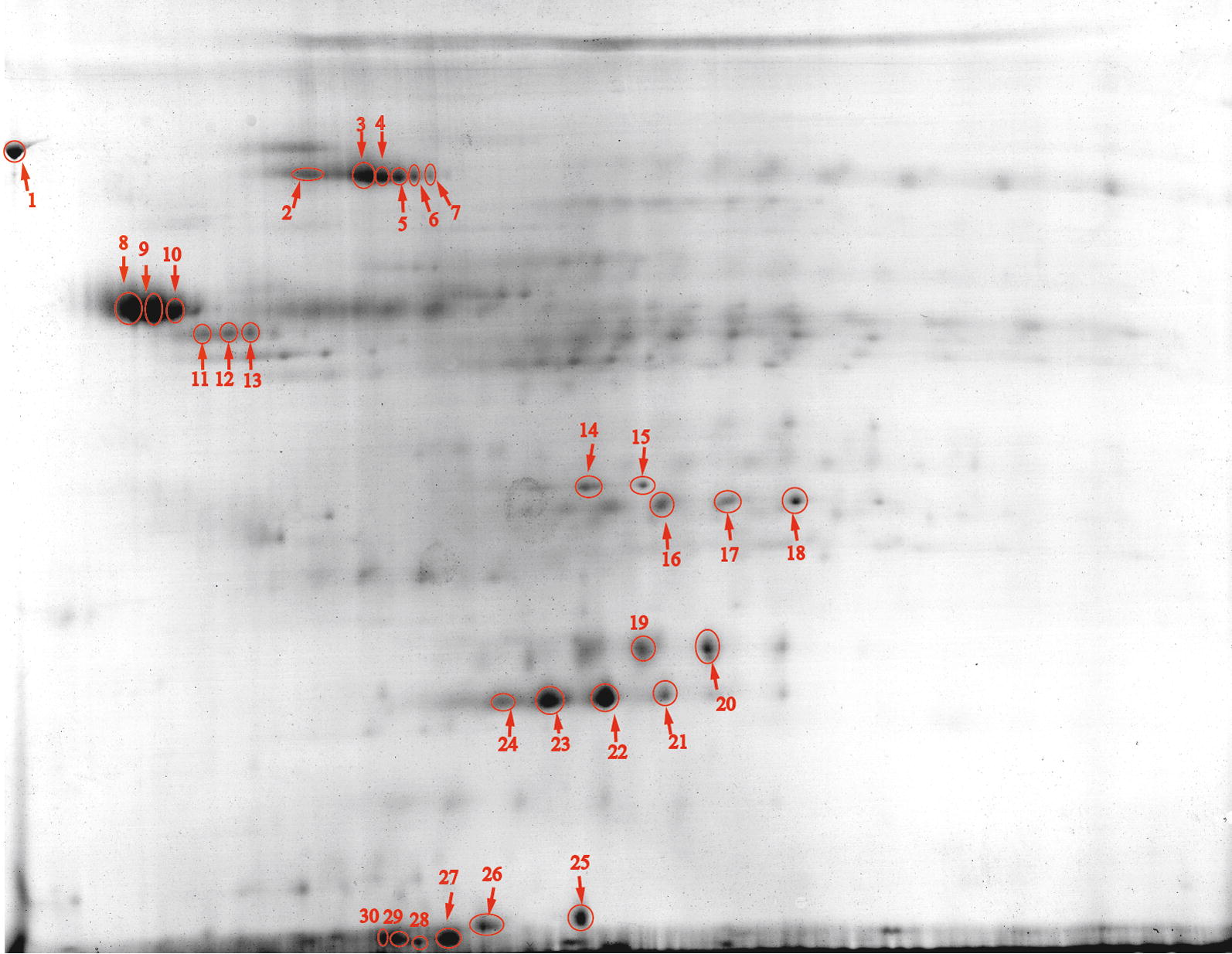
Spots of biofilm proteins on two-dimensional gel electrophoresis. Major protein spots (and their gene locus) are: 1, predicted membrane protein (CA_C3309); 2–7, chaperone GroEL (CA_C2703); 8–10, putative S-layer protein (CEA_G3563); 11–13, extracellular neutral metalloprotease NPRE (CA_C2517); 14–15, electron-transfer flavoprotein, etfB (CA_C2710); 16–18, fructose-bisphosphate aldolase (CA_C0827); 19–24, rubrerythrin (CA_C3597); 25-26, chaperone GroES (CA_C2704); 27, not know (failed); 28, cold shock protein (CA_C2990); 29–30, glyceraldehyde-3-phosphate dehydrogenase GapC (CA_C0709)

Proteins extracted from the *C. acetobutylicum* biofilm were identified by LC–MS/MS. The proteins were next ranked according to their emPAI (exponentially modified protein abundance index) which reflects their relative abundance [30]. Table 2 lists the Top 30 abundant proteins of *C. acetobutylicum* biofilm. Strikingly, most of the proteins are commonly known as physiological process related proteins, especially the molecular chaperones and stress proteins. The three most abundant proteins were GroEL, surface layer (S-layer) protein and rubrerythrin, which typically functions as molecular chaperone, structure protein and oxidative stress protein, respectively. Surprisingly, many of the proteins are typically known as intracellular proteins such as the enzymes normally functioning in central metabolism, glyceraldehyde-3-phosphate dehydrogenase (GAPDH), triose phosphate isomerase, pyruvate: ferredoxin oxidoreductase, electron transfer flavoprotein and alcohol dehydrogenase. Meanwhile, the biofilm proteins were isolated by 2D gel electrophoresis and major protein spots were identified by MALDI TOF/TOF mass spectrometry. The major proteins identified on 2D gel were well included in the Top 30 abundant proteins identified by LC–MS/MS, and the spots of the three most abundant proteins, GroEL, S-layer protein and rubrerythrin were indeed the most distinct protein spots on 2D gel (Fig. 6).

**Table 2:** Top 30 extracellular proteins in *C. acetobutylicum* biofilm identified by LC–MS/MS

	Gene locus	Score^a^	Mass^b^	Matches^c^	Sequences^d^	emPAI^e^	Description
1	CA_C2703	10,961	58,166	408	29	12.3	Molecular chaperone GroEL (Hsp60)
2	CEA_G3563	3556	47,277	139	17	10.3	Putative S-layer protein
3	CA_C3597	8141	20,493	187	10	10.3	Rubrerythrin
4	CA_C2710	2299	28,089	71	14	9.5	Electron transfer flavoprotein beta-subunit
5	CA_C0709	1619	35,999	72	15	8.1	Glyceraldehyde-3-phosphate dehydrogenase
6	CA_C2452	572	15,611	24	7	7.7	Flavodoxin
7	CA_C1555	2446	29,503	80	10	7.5	Flagellin
8	CA_C1747	556	8602	21	3	6.9	Acyl carrier protein, ACP
9	CA_C3136	5404	43,482	207	18	6.8	Elongation Factor Tu (Ef-Tu)
10	CA_C2990	396	7307	23	2	6.2	Cold shock protein
11	CA_C1834	448	9203	19	4	5.9	Host factor I protein Hfq
12	CA_C3125	299	7908	7	3	5.4	Ribosomal protein L29
13	CA_C2712	571	28,400	28	10	4.9	Crotonase
14	CA_C1807	182	10,251	7	5	4.8	Ribosomal Protein S15
15	CA_C3211	1113	10,341	59	6	4.7	DNA binding protein HU
16	CA_P0164	1098	23,666	33	8	4.6	Acetoacetyl-CoA:acetate/butyrate CoA-transferase subunit B
17	CA_C2704	745	10,420	27	5	4.6	Molecular chaperone groES (Hsp10, Hsp60 cofactor)
18	CA_C3145	553	12,670	24	5	4.4	Ribosomal protein L7/L12
19	CA_C3076	627	32,321	30	12	4.3	Phosphate butyryltransferase
20	CA_C1281	757	17,734	29	7	3.8	Heat shock protein grpE (hsp20, Hsp70 cofactor)
21	CA_C1282	2280	65,723	77	24	3.8	Molecular chaperone DnaK (Hsp70)
22	CA_C2229	4951	129,740	191	43	3.7	Pyruvate:ferredoxin oxidoreductase
23	CA_C2641	782	49,565	37	19	3.7	FKBP-type peptidyl-prolyl cis-transisomerase (trigger factor)
24	CA_C3075	744	39,146	33	15	3.7	Butyrate kinase, BUK
25	CA_C2873	1247	41,443	53	16	3.7	Acetyl coenzyme A acetyltransferase (thiolase)
26	CA_P0165	725	23,797	22	8	3.3	Acetoacetate decarboxylase
27	CA_P0162	3768	95,774	175	32	3.2	Alcohol dehydrogenase E
28	CA_C0711	452	26,698	17	11	3.1	Triosephosphate isomerase
29	CA_C2597	461	17,599	19	5	3.1	Hypothetical protein
30	CA_C3558	1128	48,599	51	10	3.0	Probable S-layer protein

^a^All scores were statistically significant (*p* < 0.05; Student t test). Higher score means higher probability

^b^Theoretical molecular mass

^c^The number of peptides that matched the identified protein with* p* < 0.05

^d^The number of distinct (nonredundant) peptides that matched the identified protein with* p *< 0.05 (Student t test)

^e^Exponentially modified Protein Abundance Index

In fact, most of the *C. acetobutylicum* biofilm proteins identified here have been recognized as a kind of non-classically secreted proteins that do not contain known sequence motifs for secretion or anchoring onto the cell surface [31, 32]. A great number of these proteins have been found to be “moonlight proteins” that have a canonical biochemical function inside the cell and perform a second biochemical function on the cell surface or extracellularly [33, 34]. Table 3 lists the proteins (from the Top 30 abundant proteins list) that have been reported as non-classically secreted proteins or moonlighting proteins. Obviously, many proteins with canonical function in central metabolism, chaperone activity, or protein synthesis and nucleic acid stability could moonlight as bacterial adhesins and interact with the environment. While these proteins were abundant in the biofilm, no apparent regulation of their gene transcription was observed (Additional file 2: Sheet 3).

**Table 3:** Major *C. acetobutylicum* biofilm proteins that have been reported as non-classically secreted proteins with potential moonlighting functions

Intracellular function	Moonlighting function
Chaperones	
Molecular chaperone groel	Adhesin [33, 35–37]; bind mucin, invertase and fibronectin [38, 39]
Molecular chaperone dnak	Bind plasminogen and invertase [40–42]
Heat shock protein grpe	Not characterized [32, 39, 43]
Molecular chaperone groes	Not characterized [32, 43]
Cold shock protein	Not characterized [44, 45]
Protein synthesis and nucleic acid stability	
Elongation factor Tu (Ef-Tu)	Attach to human cells, bind fibronectin and plasminogen [46–49]
Trigger factor	Not characterized [43, 44, 50, 51]
Ribosomal protein L29	Not characterized [43, 45, 51]
Ribosomal protein S15	Not characterized [52, 53]
Ribosomal protein L7/L12	Not characterized [52, 54]
Central metabolism	
Glyceraldehyde-3-phosphate dehydrogenase (GAPDH)	Adhesin [55–57]; bind plasminogen, collagen, fibronectin and RNA [34, 58, 59]
Triosephosphate isomerase	Adhesin [33, 60]; bind plasminogen, laminin and fibronectin [61, 62]
Alcohol dehydrogenase	Bind plasminogen [63, 64]
Pyruvate: ferredoxin oxidoreductase	Adhesin [65, 66]
Electron transfer flavoprotein beta-subunit	Not characterized [44, 50, 54]
Acetyl coenzyme A acetyltransferase (thiolase)	Not characterized [44, 67]
Rubrerythrin	Not characterized [52, 68]
Acyl carrier protein, ACP	Not characterized [69, 70]

## Discussion

*C. acetobutylicum* has attracted considerable interest due to its unique capability of biosynthesizing a range of liquid fuels and bulk chemicals that are fundamentally important to human society. It has been long accepted that sporulating clostridial form of *C. acetobutylicum* cells is the solvent-forming phenotype, that is, solventogenesis is coupled to sporulation. However, Tracy and his co-workers observed a stronger correlation between solvent production and the vegetative cell type than the clostridial-form type based on flow cytometry assisted cell-sorting techniques. They also demonstrated that a *sigF* mutant blocked sporulation but still produced comparable solvent in batch fermentation [71, 72]. Despite this, the view that solventogenesis is coupled to sporulation is still prevailing in the field [73, 74]. Here, our results clearly showed that *C. acetobutylicum* could eliminate sporulation and display vegetative growth in biofilm over time. In this way, instead of being impaired, the solvent production was greatly improved [12, 22]. Therefore, it is plausible that sporulation and solventogenesis can be uncoupled in *C. acetobutylicum*. This is of particular importance, because it would encourage researchers to develop long-term continuous cultivation processes. Besides elimination of sporulation, *C. acetobutylicum* biofilm cells also exhibited significant morphological changes. The prolonged chain-like morphology observed for *C. acetobutylicum* biofilm cells was also observed for *Bacillus subtilis* biofilm cells. In *B. subtilis*, a transcriptional regulator SinR represses the genes responsible for EPS production and promotes cell separation and motility. During biofilm development, SinR activity is antagonized. Low SinR activity results in EPS production and loss of cell motility. Thus, motile single cells switch to long chains of nonmotile cells [75]. Considering the presence of SinR in *C. acetobutylicum*, this is probably also the case in *C. acetobutylicum*.

Despite the fact that *C. acetobutylicum* biofilm has been extensively exploited for producing industrial products [4, 12, 16, 21], the biosynthetic process and molecular composition of *C. acetobutylicum* biofilm remain completely unknown. Here, for the first time isolated polysaccharides and proteins from *C. acetobutylicum* biofilm were reported. *C. acetobutylicum* biofilm contained three polysaccharides which were all heteropolysaccharides. Recently, a polysaccharide separated from *C. acetobutylicum* culture supernatant was reported [76]. Consistent with our results, the supernatant polysaccharide was also a heteropolysaccharide and its monosaccharide composition seemed similar to those of the SM2 and SM3. While the supernatant polysaccharide was characterized with glucose (34%, molar ratio), rhamnose (40%), mannose (13%) and galactose (10%) as its primary monosaccharides, the SM2 and SM3 characterized here also consisted of glucose (47–53%), rhamnose (10–16%), mannose (13–21%) and galactose (9–10%) as their primary monosaccharides (Table 1), although the monosaccharide ratio differed. However, the polysaccharide SM1 that represented the major polysaccharide in *C. acetobutylicum* biofilm had a more distinct composition: it contained predominantly glucose (58%), mannose (21%), and aminoglucose (13%). Altogether, it seemed that *C. acetobutylicum* liked to produce a variety of heteropolysaccharides varying in monosaccharide composition. In addition, the biofilm polysaccharides, especially the SM1, proved hard to re-dissolve after lyophilization. Also, they were possibly associated with some non-carbohydrate substances. Despite our try of various protein removal methods, they still defied ^1^H-NMR analysis.

Strikingly, a great variety of proteins were found abundantly present in *C. acetobutylicum* biofilm. One of the most abundant proteins was a protein annotated as putative S-layer protein (Table 2; Fig. 6). The gene encoding this protein is designated CEA_G3563 in *C. acetobutylicum* EA2018 and SMB_G3598 in *C. acetobutylicum* DSM 1731. In both strains, it is located in an operon together with and downstream of an S-layer protein encoding gene [77]. It shows 81% sequence identity to a S-layer protein from *C. felsineum* DSM 794 (Sequence ID: WP_077894211), but both have been poorly studied. S-layers are crystalline monomolecular assemblies of protein or glycoprotein, which represent one of the most common cell envelope structures in bacteria [78]. In *Clostridium difficile*, S-layer proteins were demonstrated essential for biofilm formation perhaps due to the fact that S-layer is essential for anchoring cell wall associated proteins that are required for adhesion during biofilm formation [79]. Studies also showed that S-layer was required for normal growth in *C. difficile* [80, 81], while a non-matured S-layer protein induced the apparition of a bigger biofilm [82]. Except the putative S-layer protein, most of the *C. acetobutylicum* proteins were typically intracellular proteins (Tables 2, 3). It has previously been reported that a variety of Gram-positive bacteria, such as *S. aureus* and *B. subtilis*, release intracellular proteins into the external environment during stationary phase [83, 84]. These proteins are considered to be secreted in a non-classical pathway and some of them (e.g., the GroEL) have been extensively found to moonlight as adhesins and contribute to the biofilm formation [32, 33, 84]. For instance, it was shown that deletion of GroEL-phosphorylating PrkC in *Bacillus anthracis* abrogated biofilm formation, while overexpression of GroEL led to increased biofilm formation [85, 86]. Another intracellular protein abundant in the biofilm is a rubrerythrin encoded by *rbr3B* (CA_C3597). In this rubrerythrin, the order of the two functional domains is reversed compared to normal rubrerythrins [87]. Although this rubrerythrin has been demonstrated to be involved in H_2_O_2_ and O_2_ detoxification, its role in biofilm remains to be studied.

Besides the non-classical secretion, the abundance of intracellular proteins could also be a result of cell lysis inside the biofilm during long-term development. While a biofilm could persistently exist, a subpopulation of the cells inside is likely lysed due to various mechanisms [88]. The biofilm matrix could act as a recycling center by keeping the components of lysed cells available [19, 88]. A biofilm-forming mechanism was recently proposed using *Staphylococcus aureus* or *Pseudomonas aeruginosa* biofilm as a model [83, 89]. In these models, cytoplasmic proteins that were released from cells or cell lysate proteins could associate with the cell surface in response to decreasing pH during biofilm formation. Considering the presence of abundant cytoplasmic proteins in the *C. acetobutylicum* biofilm as well as a low pH level (usually around pH 4.2) during *C. acetobutylicum* fermentation, this mechanism also be plausible for *C. acetobutylicum* biofilm.

## Conclusions

*Clostridium acetobutylicum* biofilm cells eliminated sporulation and performed vegetative growth over time, indicating that vegetative *C. acetobutylicum* cells rather than the spore-forming cells were the solvent-forming cells. EPS and wire-like structures were observed. The biofilm contained three heteropolysaccharides. The major fraction consisted of predominantly glucose, mannose and aminoglucose. A variety of proteins including non-classically secreted proteins were present in the biofilm, with GroEL, a S-layer protein and rubrerythrin being the most abundant. Of these proteins, many proteins such as GroEL, Ef-Tu and glyceraldehyde-3-phosphate dehydrogenase could moonlight as adhesins which might contribute to the biofilm formation. This study provides important insights into *C. acetobutylicum* biofilm. Future studies should genetically manipulate the main components to elucidate their specific roles in *C. acetobutylicum* biofilm.

## Additional files

**Additional file 1.** Evaluation of different extraction methods and ^1^H-NMR spectra of polysaccharides.

**Additional file 2.** Full list of the biofilm proteins and relevant gene expression data.

## Abbreviations

2D: two-dimensional
EPS: extracellular polymeric substances
LC–MS/MS: liquid chromatography coupled with tandem mass spectrometry
MALDI TOF/TOF: matrix-assisted laser desorption/ionization time-of-flight/time-of-flight mass spectrometer
PMP: 1-phenyl-3-methyl-5-pyrazolone
QFF: q-sepharose fast flow chromatography column
SDS-PAGE: sodium dodecyl sulfate–polyacrylamide gel electrophoresis
