# Supplementary material for: Clostridium acetobutylicum grows vegetatively in a biofilm rich in heteropolysaccharides and cytoplasmic proteins
Source: Biotechnol Biofuels. 2018 Nov 20;11:315. doi: 10.1186/s13068-018-1316-4 (PMC6245871; doi:10.1186/s13068-018-1316-4)
Supplement: Supplementary file 1 — Additional file 1. Evaluation of different extraction methods and 1H-NMR spectra of polysaccharides. [file 13068_2018_1316_MOESM1_ESM.docx]

**Additional file 1**


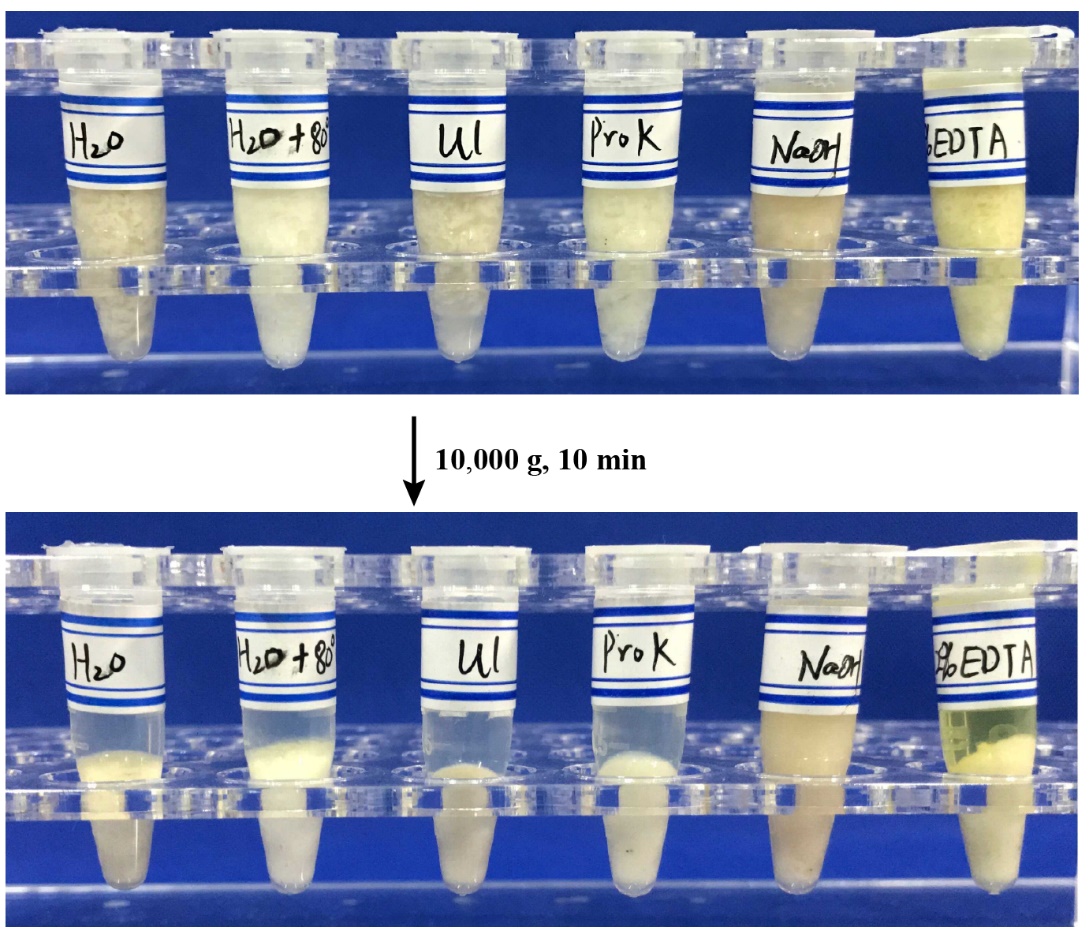


**FigureS1** *C. acetobutylicum* biofilm EPS extraction by different methods. Extractions were conducted as previously described [[1](#_ENREF_1)]. For each extraction, 0.5 g of wet biofilm (equals to 40.5 mg dry weight) was mixed with 1 ml of corresponding solution and then treated as follows: H_2_O, mixed solely with water at 25℃ for 2 h (as a control); H_2_O+80℃, heated in water at 80℃ for 2 h; Ul, ultrasonicated at 50 W for 30 min; ProK, incubated with 1 g/L of proteinase K at 37℃ for 10 h; NaOH, mixed with 0.1 M NaOH and kept at 4℃ for 30 min; EDTA, incubated with 5% EDTA for 10 h. Experiments were conducted at a shaking speed of 120 rpm. The top panel shows the biofilm suspensions after treatment by different extraction methods; the bottom panel show the biofilm pellets after further centrifugation.

**Table S1** EPS contents extracted by different methods^a^

| **Extraction methods**^b^ | **Protein (mg/g)** | **Polysaccharide (mg/g)** | **DNA (mg/g)** | **DNA ratio**^c^ |
| --- | --- | --- | --- | --- |
| 80℃ H_2_O | 13.5 ± 4.52 | 32.5 ± 9.18 | 2.75 ± 1.12 | 5.6% |
| Ultrasonication | 25.3 ± 6.13 | 45.8 ± 3.39 | 4.02 ± 2.33 | 5.4% |
| EDTA | 30.0 ± 3.45 | 65.3 ± 4.75 | 17.3 ± 4.22 | 15.4% |
| Proteinase K | 9.50 ± 5.16 | 77.5 ± 8.22 | 28.5 ± 6.02 | 24.7% |
| 0.1M NaOH | 315 ± 22.1 | 163 ± 11.9 | 46.0 ± 5.52 | 8.8% |

^a^Values are mg per g of biofilm dry weight. DNA content was measured using the diphenylamine colorimetric method [[2](#_ENREF_2)]. Protein content was determined using Coomassie brilliant blue G250 [[3](#_ENREF_3)] and polyshaccride content was determined using phenol-sulfuric acid [[4](#_ENREF_4)].

^b^Extraction methods are the same as those discribed in Figure S1.

^c^The ratio of DNA (mg/g) to the total amount of protein, polysaccharide and DNA (mg/g).


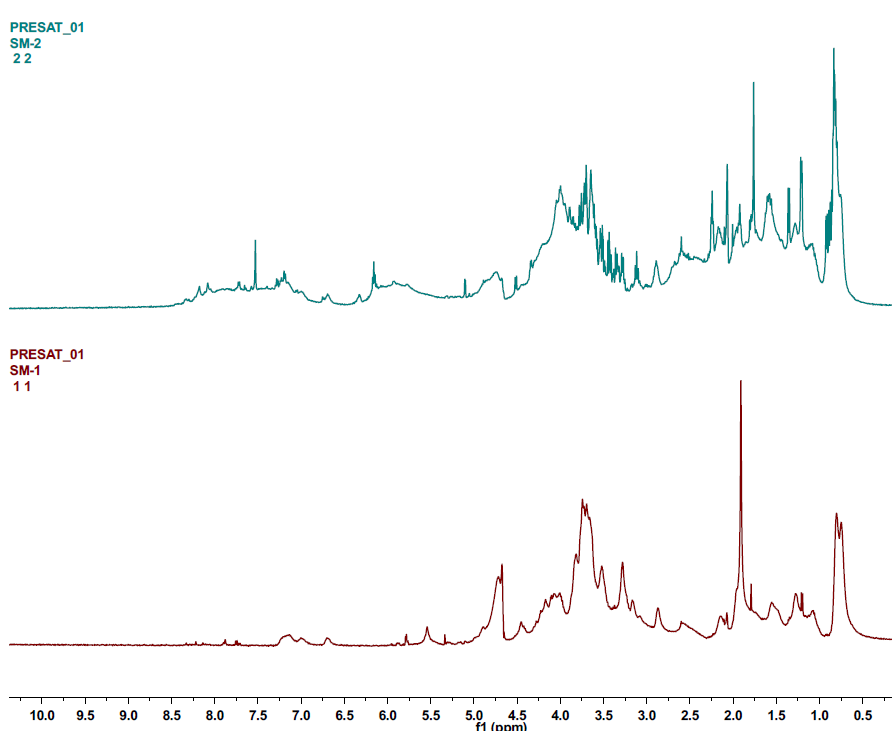


**Figure S2** ^1^H-NMR spectra of polysaccharides SM1 (bottom) and SM2 (top) isolated from *C.acetobbutylicum* biofilm with a Q-Sepharose chromatography column. Samples (40 mg) were deuterium exchanged using D_2_O three times and then dissolved in 0.5 mL of D_2_O with 35 uL of deuterated acetone. Spectra were recorded at 25 °C on an Agilent DD2 500 MHz. Analysis is difficult due to the noise pattern in the region from 0.5-5.5 ppm.

**References**

1. Sun M, Li W-W, Yu H-Q, Harada H: **A novel integrated approach to quantitatively evaluate the efficiency of extracellular polymeric substances (EPS) extraction process.** *Applied microbiology and biotechnology* 2012, **96:**1577-1585.

2. Burton K: **A study of the conditions and mechanism of the diphenylamine reaction for the colorimetric estimation of deoxyribonucleic acid.** *Biochemical journal* 1956, **62:**315.

3. Sedmak JJ, Grossberg SE: **A rapid, sensitive, and versatile assay for protein using Coomassie brilliant blue G250.** *Analytical biochemistry* 1977, **79:**544-552.

4. Dubois M, Gilles KA, Hamilton JK, Rebers Pt, Smith F: **Colorimetric method for determination of sugars and related substances.** *Analytical chemistry* 1956, **28:**350-356.
